# A case report of systemic lupus erythematosus and intestinal tuberculosis with lower gastrointestinal bleeding: A treatment approach utilizing parenteral nutrition

**DOI:** 10.1097/MD.0000000000035374

**Published:** 2023-10-13

**Authors:** Aiping Zhou, Ying Wang, Yanling Chen, Hua Zhong, Bo Chen, Chunyu Tan

**Affiliations:** a West China School of Nursing, Sichuan University/Department of Rheumatism and Immunology, West China Hospital, Sichuan University, Chengdu, China; b West China Hospital, Sichuan University/West China School of Nursing, Sichuan University, Chengdu, China; c Department of Rheumatism and Immunology, West China Hospital of Sichuan University, Chengdu, Sichuan, China.

**Keywords:** case report, intestinal tuberculosis, lower gastrointestinal bleeding, nursing, parenteral nutrition, systemic lupus erythematosus

## Abstract

**Rationale:**

Limited literatures are available on lower gastrointestinal bleeding in systemic lupus erythematosus (SLE) combined with intestinal tuberculosis. Sharing the treatment experiences of a 26-year-old female patient diagnosed with this complex condition in this report may contribute valuable insights.

**Patient concerns:**

The patient initially presented with abdominal pain and active gastrointestinal bleeding, leading to admission to the hospital. Over a 2-week period, she experienced persistent bleeding, with daily volumes ranging from 300 mL to 800 mL.

**Diagnoses:**

Lower gastrointestinal bleeding was diagnosed in this patient with concurrent systemic lupus erythematosus and intestinal tuberculosis.

**Interventions:**

As her symptoms rapidly progressed, food and water intake had to be completely restricted. The parenteral nutrition was implemented.

**Outcomes:**

The medical team effectively controlled the bleeding, leading to a notable improvement in the patient’s condition. Consequently, she was able to resume oral intake and was discharged from the hospital.

**Lessons:**

This case highlights the significance of using parenteral nutrition in the management of lower gastrointestinal bleeding in patients with concurrent systemic lupus erythematosus and intestinal tuberculosis. Close monitoring and collaborative efforts among healthcare professionals are crucial to achieve successful outcomes in similar cases.

## 1. Introduction

Systemic lupus erythematosus (SLE) is a commonly observed autoimmune disease that primarily affects the digestive system. It manifests in various systems and organs, particularly the gastrointestinal tract and small intestine.^[1]^ Asian populations face a higher risk of mortality associated with SLE compared to other populations.^[2,3]^ The compromised immune function and long-term immunosuppressive therapy in SLE patients increase their susceptibility to infections.^[4]^ Consequently, the incidence of opportunistic infections, including tuberculosis (TB), is higher in SLE patients than in the general population.^[5]^ Extrapulmonary TB, which includes intestinal TB, accounts for a significant proportion of TB infections in China.^[6]^

The risk of TB may be increased by autoimmune illnesses like SLE, particularly when immunosuppressive drugs are required due to disease activity. According to earlier research^[7,8]^ using steroids or immunosuppressive medications implies significant potential risk factors for infections. The mainstay of SLE treatment is an immunosuppressive drug regimen including corticosteroids. In Colombia, retrospective case-control research revealed that a 12-month cumulative steroid dose of <1830 mg nearly increased the risk of TB.^[9]^ In a cohort study conducted in Taiwan, cyclophosphamide was linked to an almost 3-fold increased risk of TB.^[10]^

Lower gastrointestinal bleeding, which refers to bleeding from the gastrointestinal tract below the Treitz ligament, constitutes around 20% of all gastrointestinal bleeding cases.^[11]^ In patients with systemic lupus erythematosus (SLE) combined with gastrointestinal tuberculosis, lower GI bleeding is a rare yet significant complication. Patients with ongoing lower GI bleeding often need to refrain from consuming food and liquids. Unfortunately, TB is a wasting disease, and adequate nutrition plays a crucial role in disease recovery. Parenteral nutrition (PN) is administered when patients are unable to receive nutrition through the gastrointestinal tract and when they have significant gastrointestinal diseases. PN includes total parenteral nutrition (TPN) and supplemental PN. Currently, there is a notable scarcity of reports on the clinical nutrition treatment of patients with these combined conditions. In March 2022, a patient with SLE combined with intestinal TB and lower gastrointestinal bleeding was admitted to the Department of Rheumatology and Immunology at our hospital. In this case report, we present the care experience of this patient.

## 2. Case presentation

### 2.1. Clinical information

The patient is a 26-year-old female who presented with the following symptoms: fever up to 39°C, general malaise, poor respiratory function, wasting, depressed edema of both lower limbs, and chest tightness and shortness of breath after activity. Blood tests revealed the following results: anti-nuclear antibody + 1:100 granular, anti-RNP antibody ++, anti-SSA antibody ++, anti-R0–52 antibody ++, and anti-ribosomal P protein antibody ++. Venous ultrasound of both lower limbs showed bilateral common femoral vein thrombosis. The patient had received treatment with injectable methylprednisolone sodium succinate and low-molecular heparin sodium injection prior to admission. Anticoagulant drugs were discontinued half a month ago due to abdominal pain and blood in the stool. On March 18, 2022, she was admitted to our department following an emergency department visit.

### 2.2. Examination

The patient presented with the following findings: mental clarity, poor spirit, anemia, emaciation, generalized puffiness, marked right lower abdominal pressure pain, positive active turbid sounds, severe depressed edema of both lower limbs, grade 3 muscle strength of both lower limbs, marked chest tightness, and shortness of breath after activity. Initial blood tests on admission showed an anti-double-stranded DNA antibody level of 130.00 IU/mL (chemiluminescence or immunofluorescence method), positive anti-Sm antibody, and complement C3 level of 0.431g/L. Vascular ultrasound revealed thrombosis and partial recanalization of the left common iliac vein and bilateral external lower extremity veins, as well as a large intra-abdominal fluid collection. A chest CT scan showed scattered inflammation in both lungs, a small amount of pericardial effusion, and fluid compression of lung tissue in adjacent chest cavities. An abdominal CT scan with contrast enhancement revealed multiple nodules in the abdominal cavity and retroperitoneum, with partial fusion, heterogeneous enhancement, circumferential enhancement, and enlarged lymph nodes. Despite recurrent blood in the stool, the patient general condition improved over time. On April 8, 2022, a painless colonoscopy revealed multiple ulcerative resection lesions in the drawbar resection, transverse colon, splenic flexure, descending colon, rectum, and ileocecal fistula. A positive fecal Xpert test was performed on April 11, 2022, leading to the administration of levofloxacin, isoniazid, rifampin, ethambutol, and pyrazinamide. The patient was eventually discharged after correction of electrolyte disturbance.

### 2.3. Diagnosis

Upon admission, the physician promptly diagnosed the patient with systemic lupus erythematosus (SLE) with gastrointestinal bleeding, based on the 2019 EULAR/ACR grading criteria,^[12]^ as well as pre-admission findings and post-admission clinical presentation. Despite hormone and immunosuppressive treatment, the patient experienced recurrent gastrointestinal bleeding and ongoing SLE activity. A stool reexamination revealed a positive result for Xpert. The patient received concomitant hormonal, immunosuppressive, and TPN supportive therapy. After initiating antituberculosis therapy, the disease was rapidly controlled, and there was no further bleeding in the gastrointestinal tract.

### 2.4. Interventions

#### 2.4.1. Joint management of multidisciplinary teams building a multidisciplinary team.

After the patient was admitted to the hospital, she was placed on fasting and fluid restrictions due to gastrointestinal bleeding. During the course of treatment, the patient was diagnosed with intestinal TB, leading to complex situations such as electrolyte disorders and nutritional imbalances. Therefore, a multidisciplinary collaborative treatment model was adopted for this patient by the department.

A disease management team was established, consisting of the rheumatology and immunology department, gastroenterology department, TB department, and cardiovascular medicine department. The team worked together to address the unique challenges faced by the patient, ensuring a comprehensive approach to care and effective coordination among specialities.

In addition, a nutrition management team was formed, which included the rheumatology and immunology department, nutrition department, and rehabilitation department. This team focused on managing the patient nutritional needs and addressing any imbalances resulting from the fasting and fluid restrictions.

Furthermore, a glucose management team was created, involving the rheumatology and immunology department and the endocrinology department. This team collaborated to monitor and regulate the patient blood glucose levels, taking into consideration the specific requirements arising from the treatment and management of the patient conditions.

#### 2.4.2. Formulating the TPN diagnosis, treatment and care plan.

During the course of treatment, the patient experienced significant fluctuations in blood sugar levels. To address this, the multidisciplinary team engaged in frequent offline meetings and discussions, actively adjusting the treatment plan. The team closely monitored the patient vital signs and daily indices through a WeChat group, allowing for real-time evaluation and intervention. In cases where the gastrointestinal tract is damaged, and enteral nutrition is not feasible, an individualized PN program was developed, tailored specifically to the patient needs. A rheumatology immune nurse played a pivotal role in implementing the program, promptly reporting any issues during TPN treatment to the physician and optimizing the personalized care plan accordingly.

#### 2.4.3. Personalized volume management during TPN treatment.

To ensure individualized volume management, a collaborative approach involving physicians and charge nurses was implemented. Daily morning and evening examinations were conducted, and blood pressure and volume management goals were established. The target blood pressure range was set at 80 to 100/50 to 70 mm Hg, and the target heart rate ranged between 60 and 100 beats per minute. Bedside cardiac monitoring was utilized to increase the frequency of blood pressure and heart rate monitoring. Accurate recording of the patient 24-hour intake and output was performed, including observation of stool color, nature, and volume. Stool color was recorded using a cell phone, and volume was measured accurately with a measuring cup and recorded.

Personalized TPN treatment based on the patient weight and daily fluid intake and output (45 kg) was provided. The patient daily nutritional fluid volume was calculated (1417 mL). The amount of nutritional and medication fluids was adjusted based on the patient changing condition, ensuring that the total daily intravenous fluid input did not exceed 3000 mL per day. TPN therapy was strictly controlled by adjusting the intravenous drip rate to 100 mL/hour.

The nurse daily measured, observed, and recorded the regression of bilateral lower extremity sunken edema. To improve cardiac volume load and reduce blood pressure fluctuations, hydrocoagulants were administered in small doses via intravenous infusion, as ordered by the physician.^[13]^ Close monitoring was conducted to prevent electrolyte disorders such as hypokalemia and hyponatremia, and the patient was observed for symptoms related to mental status, abdominal distention, and cardiac arrhythmias.

#### 2.4.4. Adequate medical and nursing cooperation prior to TPN treatment Preparation of nutritional fluids.

In the case of our patient, a nutritional risk screening was conducted, and the patient received a score of 16. The dietitian determined that the patient required tertiary nutrition due to lower gastrointestinal bleeding, necessitating fasting. Consequently, a total nutrient admixture (TNA) was employed for clinical nutrition therapy. Studies have demonstrated that the “total nutrition mix” offers complete nutrients that can be adjusted to meet the individualized treatment needs of patients.^[14]^ Our hospital utilizes an in-house “all-in-one” nutrition solution, providing a comprehensive range of nutrients that can be flexibly and promptly adjusted based on the patient evolving condition. After the physicians prescribe the PN, the request undergoes review by the pharmacist and is prepared by the intravenous dispensing center. Once the nutrition solution is prepared and stored in a light-protected environment, it is delivered to the ward for TPN treatment by the hospital transport and distribution department.

#### 2.4.5. Expert nurse line TPN treatment pipeline preparation.

After discussing the patient condition with the nurse and the nursing venipuncture center, it was decided to use a temporary central venous catheter through subclavian vein puncture upon the patient arrival at the hospital. Following repeated communication with the patient and their family, the nurse inserted the central venous catheter through a peripheral vein using the expertise of 2 specialist nurses. To ensure the proper placement of the central venous catheter and prevent TNA nutrient fluid from entering the pleural cavity, a bedside chest x-ray was performed by a technician before initiating TPN therapy. The x-ray helped determine the location of the catheter tip, which was positioned at the lower 1/3 of the connection between the superior vena cava and the right atrium.^[15]^ With careful placement, the catheter was successfully positioned and ready for use, ensuring patient safety.

#### 2.4.6. Nurse involvement in glycemic management during TPN treatment.

##### 2.4.6.1. Management of hyperglycemia.

The following measures were taken to address hyperglycemia in the patient:

1. Close monitoring of glycemic changes: Blood glucose was monitored every 4 to 6 hours during TPN treatment. If hyperglycemia occurred, the nurse increased the frequency of blood glucose monitoring to every hour until the blood glucose value approached the relaxed range of the target blood glucose value, at which point monitoring was stopped.^[16]^ During the first week of the patient TPN treatment, the nurse observed that the patient was prone to hypoglycemic reactions such as panic and sweating in the early morning and that the symptoms were not easily detected. After consultation with the glucose management team, the nurse changed the glucose monitoring to one where the patient blood glucose values were within a loose range, and then monitored the blood glucose at 3:00 am and the change in blood glucose at 7:00 am. In the second week after the patient received TPN treatment, the nurse found that the patient had a hypoglycemic reaction in the early morning and performed hypoglycemia management, and the patient nighttime hypoglycemia was better improved; Internet-based professional consultation management model: Nurses monitored the patient blood glucose levels and reported them to the inpatient Internet management system. High and low blood glucose values were promptly reported through the system, allowing for timely diagnosis and treatment by the blood glucose management team; some studies have shown that growth inhibitors can delay the absorption of blood glucose and inhibit the secretion of glucagon and insulin.^[17]^

These measures aimed to optimize blood glucose control and ensure the patient safety and well-being during TPN therapy:

1. Observing patients stools daily is an important nursing practice to monitor lower gastrointestinal bleeding. In the case of this patient, several measures were taken to address and stabilize their blood glucose levels:
2. Discontinuation and reduction of growth inhibitor: The growth inhibitor was discontinued, and the rate and dose of the growth inhibitor pump were reduced to gradually stabilize the patient blood glucose levels.
3. Insulin administration via intravenous micropump: When the patient blood glucose level was measured as HI (high, April 2), the nurse used a single intravenous micropump to administer insulin diluent. The pumping speed of insulin was adjusted according to the patient blood glucose fluctuations. After observing a significant drop in blood glucose levels during monitoring, the insulin diluent pumping was stopped when it approached the target value for blood glucose relaxation.
4. Subcutaneous injection of short-acting insulin: On a later date, the nurse administered short-acting insulin subcutaneously. However, after monitoring the patient blood glucose levels, it was observed that the levels remained high (23.7 mmol/L, April 4). Another subcutaneous injection of short-acting insulin was given, and after monitoring for an hour, the blood glucose dropped to a more acceptable level (14.9 mmol/L). The fluctuation in blood glucose levels was attributed to the interruption of continuous TPN infusion during the patient examination and the delay in timely preparation of nutrition fluid by the nutrition fluid preparation center.^[18]^
5. Continuous 24-hour infusion of nutrition fluid using an infusion pump: To minimize blood glucose fluctuations, it is necessary to infuse nutrition fluid continuously for 24 hours as per guidelines. The infusion rate is calculated based on the total amount of nutrition fluid and infusion time, and an infusion pump is used to control the rate and ensure consistent infusion, thus preventing rapid or inconsistent infusion that may lead to blood glucose fluctuations.
6. Use of ethylene vinyl acetate infusion bottles for insulin administration: Polyvinyl chloride infusion containers have been found to have an adsorption effect on insulin. To minimize this effect, the nurse used ethylene vinyl acetate infusion bottles for the infusion, which reduces the adsorption effect on insulin.^[19]^

By implementing these measures, the patient hyperglycemia gradually improved, and steps were taken to ensure more stable blood glucose levels during TPN therapy.

##### 2.4.6.2. Management of hypoglycemia.

During the patient treatment with TPN, their fingertip glucose levels showed fluctuations between 0.9 and 3.3 mmol/L, primarily occurring at 3 and 11 am. The dietitian recommended controlling the duration of nutrient infusion between 20 and 22 hours, resulting in a 1 to 2 hour interruption in the daily nutrient infusion. However, the patient condition of intestinal TB and high consumption state was not taken into account, and the nutritional fluids provided were inadequate to meet their needs. Furthermore, the duration of blood glucose monitoring was not adjusted promptly based on the patient changing condition. The patient had multisystem involvement, and the guidelines recommend maintaining their random glucose target within a loose range of 7.8 to 13.9 mmol/L.^[20]^

The nurse collaborated with the glucose management team to address the patient hypoglycemia by implementing the following measures. Initially, considering the patient lower gastrointestinal bleeding and fasting, in one instance of hypoglycemia, an immediate administration of 50% dextrose injection (30 mL IV push) was carried out according to the guidelines. Fifteen minutes later, a fingertip glucose test was performed, revealing a measurement of 2.3 mmol/L. To address the persisting hypoglycemia, another dose of 50% dextrose injection (60 mL IV push) was administered, and after another 15 minutes, a fingertip glucose test showed an improvement to 5.6 mmol/L.^[21]^

To ensure continuous infusion of the nutrient solution, a 24-hour infusion was scheduled. However, due to a delay of 1 to 2 hours in configuring and distributing the nutrient solution for morning hypoglycemic patients in our hospital, nurses undertook the monitoring of hypoglycemic patients in the early morning. Slow intravenous infusion of glucose injection was employed as a preventive measure against hypoglycemic symptoms.^[16]^ The use of an infusion pump was implemented to regulate the rate of nutrient solution delivery, preventing overly rapid infusion.

#### 2.4.7. Step-by-step management of nurse-dietitian collaboration to convert TPN therapy to transoral enteral nutrition.

The following specific measures were implemented with the assistance of nurses and the nutrition management team, along with diet and nutrition education:

1. The dietitian adjusted the TNA nutrition solution regimen. Initially, the patient daily TNA nutrition solution provided 1159 kcal of energy. Gradually, based on the patient enteral nutrition needs, the daily TNA nutrient solution energy was reduced to 691 kcal.
2. Enteral nutrition solution was provided 3 times a day, with each bag of nutrition solution containing 156 kcal of energy.
3. Fasting and diet were monitored after each of the 3 meals.
4. A feeding plan was developed for the patient during the transition phase between enteral nutrition and oral feeding. The nurse provided guidance on correct feeding, and the patient weight was continuously measured.^[22]^
5. It was necessary to advise the patient that in case of severe nausea, vomiting, abdominal distention, or abdominal pain, the diet plan should be adjusted accordingly, and if necessary, oral feeding should be suspended. During the TPN combined with enteral nutrition therapy, the patient did not experience any adverse effects, and she was discharged in good condition.

## 3. Discussions

### 3.1. Coordinated care through multidisciplinary teams establishing an effective multidisciplinary team

Establishing an effective multidisciplinary team requires a commitment to collaboration, effective communication, and shared decision-making. By working together, healthcare professionals can provide holistic and coordinated care that optimizes patient outcomes and enhances the overall healthcare experience. Reports of systemic lupus erythematosus (SLE) combined with intestinal TB and lower gastrointestinal bleeding are extremely rare. Throughout the entire care process, nurses played a pivotal role, actively participating in disease management, nutrition management, blood glucose management, pipeline management, and facilitating the transition from parenteral to enteral nutrition management. Their involvement ensured continuity of care and consistent support for the patient.

By implementing this comprehensive multidisciplinary approach, the department aimed to optimize the patient outcomes, effectively addressing the complex nature of SLE, intestinal TB, gastrointestinal bleeding, electrolyte disorders, nutritional imbalances, and glucose management.

### 3.2. Developing a comprehensive TPN diagnosis, treatment, and care plan

For this patient, TPN plays a crucial role in providing essential nutrients, electrolytes, and energy substrates. As the patient is unable to consume food orally due to gastrointestinal bleeding and dysfunction, TPN becomes the primary source of nutrition, sustaining the patient life, supporting nutritional needs, and enhancing the body immunity during the critical period.^[23]^

TPN is particularly beneficial in the early stages of hospitalization when the patient gastrointestinal function is compromised. It bypasses the gastrointestinal tract, reducing stimulation and minimizing the secretion of gastrointestinal mucus. By delivering adequate energy and nitrogen sources, TPN helps meet the patient nutritional requirements and promotes recovery.^[23]^ However, TPN should not be used as the sole source of nutrition for an extended period. Prolonged fasting and reliance on TPN can result in gastrointestinal dysfunction and hinder the recovery of gastrointestinal function. Recognizing this, the multidisciplinary team carefully considered the patient condition and made the decision to administer TPN.

The proactive monitoring, collaboration, and adjustments made by the multidisciplinary team and the

By following these steps and engaging in ongoing assessment, monitoring, and collaboration, healthcare providers can develop a comprehensive TPN diagnosis, treatment, and care plan that optimizes patient outcomes and ensures safe and effective TPN therapy.

#### 3.2.1. Optimizing fluid balance: personalized volume management in TPN treatment.

By prioritizing personalized volume management strategies in TPN treatment, healthcare providers can achieve optimal fluid balance, minimize the risks of fluid overload or dehydration, and enhance patient outcomes. Regular assessment, monitoring, and collaboration within the multidisciplinary team contribute to the safe and effective delivery of TPN therapy, ensuring the best possible care for patients. If left untreated, gastrointestinal bleeding can lead to severe consequences, including hemorrhagic shock and potential mortality. The initial care focus in this case is to maintain hemodynamic and circulatory stability, as well as effective volume management.^[24]^ Upon admission, the patient exhibited fluctuating blood pressure ranging between 70 and 90/40 and 60 mm Hg (1 mm Hg = 0.133 kPa) and a heart rate between 110 and 140 beats per minute. The patient had significantly reduced cardiac output, extremely low circulatory volume, and experienced chest tightness and shortness of breath even at rest in a semi-sitting position. At the beginning of admission, the cause of gastrointestinal bleeding was unclear, and the patient was placed on fasting while receiving hemostatic agents and rehydration. Clinical dietitians recommended that the nutrient fluid used in TPN treatment should not contain more than 150 mmol/L of monovalent cations (Na+, K+) and more than 10 mmol/L of divalent cations (Ca2+, Mg2+). The patient responded well to TPN treatment, and hypovolemic symptoms were successfully corrected during the hospitalization period.

### 3.3. Enhancing collaboration between medical and nursing teams for pre-treatment preparation of nutritional fluids in TPN

Prior to initiating TPN therapy, it is crucial to effectively communicate with patients, ensuring he understand the significance of TPN in their treatment plan. National and international studies highlight the importance of nutritional risk screening before commencing clinical nutrition therapy.^[25,26]^ Patients with a nutritional risk score of ≥3 tend to have better clinical outcomes following clinical nutrition therapy. Nurses on the team can employ the subjective global assessment to assess the patient nutritional status.^[27,28]^ By enhancing collaboration between the medical and nursing teams, healthcare providers can ensure the effective and safe preparation of nutritional fluids for TPN treatment. This collaborative approach promotes a shared understanding of patient needs, facilitates efficient communication, and ultimately improves the overall quality of care provided to patients receiving TPN therapy.

### 3.4. Establishing an expert nurse line for TPN treatment pipeline preparation

According to relevant guidelines, nutrition solutions with an osmolarity of <900 mmol/L can be administered through peripheral veins. However, if PN is required for more than 10 days and/or in patients with high osmolarity (≥900 mmol/L), central venous infusion is recommended.^[29]^ In the case of the patient, due to prolonged fasting, limited water intake, the use of high concentrations of potassium chloride and other medications, and an osmolarity of ≥900 mmol/L in the nutrition solution, there is a risk of irritation to the patient vasculature, and extravasation can lead to local tissue necrosis.^[30]^

By establishing an expert nurse line dedicated to TPN treatment pipeline preparation, healthcare organizations can ensure proficiency, promote patient safety, and support ongoing education and quality improvement efforts. This resource provides nurses with access to specialized knowledge, guidance, and prompt support, ultimately optimizing the preparation process and enhancing the overall quality of TPN treatment.

### 3.5. Collaborative role of nurses in glycemic management during TPN treatment

Nurse involvement in glycemic management during TPN treatment is vital for achieving optimal glycemic control, preventing complications, and promoting positive patient outcomes. Through their expertise and close monitoring, nurses contribute significantly to the overall success of TPN therapy.

#### 3.5.1. Managing hyperglycemia: strategies and approaches.

Blood glucose monitoring and control play a crucial role in clinical nutrition therapy.^[31]^ During TPN therapy, it is important to analyze the reasons behind high fingertip glucose values when the blood glucose meter shows HI (high blood glucose value ≥ 33.3 mmol/L). Several factors could contribute to elevated blood glucose levels in patients receiving TPN therapy, including: non-diabetic patients not routinely using insulin during TPN therapy; TNA formula not adjusted according to the patient condition; lack of uniform and continuous administration of the nutrient solution over 24 hours; concurrent administration of glucose water as part of other medications; adverse effects of growth inhibitors, which can lead to metabolic and nutritional disorders such as elevated blood glucose and hyperglycemia; use of daily intravenous glucocorticoids, which can affect glucose metabolism.^[32]^ Guidelines specify a target blood glucose range of 7.8 to 10.0 mmol/L for glycemic control in nutritionally supported inpatients.^[20]^ However, in the case of this patient with severe multisystem involvement, a wider range of 7.8 to 13.9 mmol/L was maintained for glycemic control.^[21,33]^ By implementing these strategies and approaches, nurses can effectively manage hyperglycemia, promote glycemic control, and improve patient outcomes. Their role in patient education, medication management, and collaborative care is essential for successful hyperglycemia management.

#### 3.5.2. Addressing hypoglycemia: effective strategies and approaches.

During the continuous infusion of the growth inhibitor dilution, the administration of the growth inhibitor was stopped promptly based on the patient gastrointestinal bleeding status. The patient blood glucose levels were monitored every 2 hours for changes and stabilized every 4 to 6 hours. Ultimately, the nurse and dietitian engaged in discussions to adjust the composition of the nutrition solution to meet the patient energy requirements. With meticulous care, the patient no longer exhibited symptoms of hypoglycemia thereafter. By implementing these effective strategies and approaches, nurses can help address hypoglycemia promptly, minimize its potential complications, and support patients in achieving and maintaining optimal glycemic control.

### 3.6. Converting TPN therapy to transoral enteral nutrition through nurse-dietitian collaboration

During the later stages of the patient hospitalization, the gastrointestinal bleeding ceased. It is crucial to consider the avoidance of energy overload during the transition from TPN therapy to enteral nutrition support therapy in clinical nutrition therapy.^[15]^ Following the normal daily nutritional requirement of 25 kcal/kg/day, with a sugar-to-fat ratio of 1:1 and non-protein calories of 100 to 200 kcal per gram of nitrogen (1gN = 6.25 g amino acids), the intake of various energy substances needs to be converted, considering the patient age, gender, height, weight, and condition. The intake should be 90% to 10% of the basic energy consumption.^[34]^ Personalized step management is essential when transitioning from TPN treatment to enteral nutrition support, taking into account the energy ratio of the 2 nutrition support regimens. By following this step-by-step approach and fostering strong nurse-dietitian collaboration, healthcare providers can facilitate a successful transition from TPN therapy to transoral enteral nutrition. This collaborative management strategy ensures patient safety, optimal nutrition, and a smooth continuum of care throughout the conversion process.

## 4. Conclusion

PN therapy for SLE combined with intestinal TB and lower gastrointestinal bleeding lacks detailed studies and relevant nursing experience. Our case involved a patient requiring extensive clinical care for long-term PN therapy. The nurse successfully provided nutritional support therapy, developed a treatment plan, administered TPN, managed blood glucose, and facilitated the transition to enteral nutrition. This experience serves as a reference for future nurses in similar cases. However, there are still deficiencies in clinical nutrition therapy, such as the need for standardized blood glucose management during TPN therapy.

## Author contributions

**Conceptualization:** Aiping Zhou.

**Data curation:** Aiping Zhou, Ying Wang, Yanling Chen, Hua Zhong, Bo Chen, Chunyu Tan.

**Formal analysis:** Ying Wang, Yanling Chen, Hua Zhong, Bo Chen, Chunyu Tan.

**Investigation:** Aiping Zhou, Ying Wang, Yanling Chen.

**Methodology:** Hua Zhong, Bo Chen, Chunyu Tan.

**Resources:** Hua Zhong.

**Supervision:** Aiping Zhou.

**Writing – original draft:** Aiping Zhou, Chunyu Tan.

**Writing – review & editing:** Aiping Zhou, Chunyu Tan.

## References

[1] Lv LG Lin YW. Emphasis on gastrointestinal involvement in systemic lupus erythematosus. Chin J Gastroenterol. 2020;40:289–91.

[2] Kinsey D Paul CP Taylor D. The whole lupus: articulating biosocial interplay in systemic lupus erythematosus epidemiology and population disparities. Health Place. 2018;51:182–8. 29655130 10.1016/j.healthplace.2018.03.007

[3] Tanaka Y O’Neill S Li M. Systemic lupus erythematosus: targeted literature review of the epidemiology, current treatment, and disease burden in the Asia Pacific region. Arthritis Care Res (Hoboken). 2022;74:187–98. 32841537 10.1002/acr.24431

[4] Barber MRW Drenkard C Falasinnu T. Global epidemiology of systemic lupus erythematosus. Nat Rev Rheumatol. 2021;17:515–32. 34345022 10.1038/s41584-021-00668-1 PMC8982275

[5] Yang Y Thumboo J Tan BH. The risk of tuberculosis in SLE patients from an Asian tertiary hospital. Rheumatol Int. 2017;37:1027–33. 28286903 10.1007/s00296-017-3696-3

[6] Mehta PK Raj A Singh N. Diagnosis of extrapulmonary tuberculosis by PCR. FEMS Immunol Med Microbiol. 2012;66:20–36. 22574812 10.1111/j.1574-695X.2012.00987.x

[7] Hamijoyo L Sahiratmadja E Ghassani NG. Tuberculosis among patients with systemic lupus erythematosus in Indonesia: a cohort study. Open Forum Infect Dis. 2022;9:ofac201. 35794932 10.1093/ofid/ofac201 PMC9251660

[8] Jung JY Yoon D Choi Y. Associated clinical factors for serious infections in patients with systemic lupus erythematosus. Sci Rep. 2019;9:9704. 31273256 10.1038/s41598-019-46039-5 PMC6609713

[9] González-Naranjo LA Coral-Enríquez JA Restrepo-Escobar M. Factors associated with active tuberculosis in Colombian patients with systemic lupus erythematosus: a case-control study. Clin Rheumatol. 2021;40:181–91. 32529420 10.1007/s10067-020-05225-x

[10] Yang SC Lai YY Huang MC. Corticosteroid dose and the risk of opportunistic infection in a national systemic lupus erythematosus cohort. Lupus. 2018;27:1819–27. 30103646 10.1177/0961203318792352

[11] Williams JG Roberts SE Ali MF. Gastroenterology services in the UK. The burden of disease, and the organisation and delivery of services for gastrointestinal and liver disorders: a review of the evidence. Gut. 2007;56(Suppl 1):1–113. 10.1136/gut.2006.117598 PMC1860005 17303614

[12] Aringer M Costenbader K Daikh D. 2019 European league against rheumatism/American college of rheumatology classification criteria for systemic lupus erythematosus. Ann Rheum Dis. 2019;78:1151–9. 31383717 10.1136/annrheumdis-2018-214819

[13] Zhu CC Ma RY. Progress in the study of volume management in patients with heart failure. Chin J Mod Nurs. 2021;27:841–5.

[14] Chen W Zhou CL Li HL. Investigation of the clinical application of parenteral nutrition premixed formulas and all-in-one liquid formulas. Chin J Gen Surg. 2011;26:762–5.

[15] Gorski LA Hadaway L Hagle ME. Infusion therapy standards of practice, 8th edition. J Infus Nurs. 2021;44:S1–S224. 33394637 10.1097/NAN.0000000000000396

[16] Yang J Zhang XW. Effect of preoperative and postoperative growth inhibitors on elderly patients with gastrointestinal bleeding. Med Rev. 2021;27:204–8.

[17] Chinese Medical Doctor Association Endocrinology and Metabolism Physician Branch; China expert group on blood sugar management of inpatients. Expert consensus on blood sugar management of inpatients in China. Chin J Endocrinol Metab. 2017;33:1–10.

[18] Zhao B Lao DH Shang YG. Standardization of parenteral nutrition solution preparation. Chin J Clin Nutr. 2018;26:136–48.

[19] Ni YH Zhang ZS Ye XH. Observation of insulin adsorption by two infusion container configurations of the nutrition solution. Parenter Enteral Nutr. 2008;15:104–6.

[20] McMahon MM Nystrom E Braunschweig C. Clinical guidelines: nutrition support of adult patients with hyperglycemia. JPEN J Parenter Enteral Nutr. 2013;37:23–36. 22753619 10.1177/0148607112452001

[21] Chinese Medical Association, Division of Diabetes. Chinese guidelines for the prevention and treatment of type 2 diabetes mellitus (2020 edition). Int J Endocrinol Metab. 2021;41:482–548.

[22] Wang Y Tian X Zhang M. Supportive care interventions for patients with home enteral nutrition after esophageal cancer surgery. J Nurs. 2021;36:96–8.

[23] Hamdan M Puckett Y. Total Parenteral Nutrition. In: StatPearls. Treasure Island (FL): StatPearls Publishing; 2023. 32644462

[24] Oakland K Guy R Uberoi R. Acute lower GI bleeding in the UK: patient characteristics, interventions and outcomes in the first nationwide audit. Gut. 2018;67:654–62. 28148540 10.1136/gutjnl-2016-313428

[25] Cederholm T Jensen GL Correia M. GLIM criteria for the diagnosis of malnutrition - a consensus report from the global clinical nutrition community. Clin Nutr. 2019;38:1–9. 30181091 10.1016/j.clnu.2018.08.002

[26] Shi HP Zhao QC Wang KH. Tertiary diagnosis of malnutrition. Chin J Cancer Control. 2015;7:313–9.

[27] Baker JP Detsky AS Wesson DE. Nutritional assessment: a comparison of clinical judgement and objective measurements. N Engl J Med. 1982;306:969–72. 6801515 10.1056/NEJM198204223061606

[28] Kondrup J Rasmussen HH Hamberg O. Nutritional risk screening (NRS 2002): a new method based on an analysis of controlled clinical trials. Clin Nutr. 2003;22:321–36. 12765673 10.1016/s0261-5614(02)00214-5

[29] Cancer N, Committee of China Anti-Cancer Association Parenteral Nutrition, Branch of Chinese Medical Association. Chinese expert consensus on the safe management of parenteral nutrition. Electron J Oncol Metab Nutr. 2021;8:495–502.

[30] Li XH Zhu LH. A case of hypokalemia caused by leakage of high concentration potassium chloride. Contemp Nurs. (last issue). 2017;6:166–8.

[31] McClave SA Taylor BE Martindale RG. Guidelines for the provision and assessment of nutrition support therapy in the adult critically ill patient: Society of Critical Care Medicine (SCCM) and American Society for Parenteral and Enteral Nutrition (A.S.P.E.N.). JPEN J Parenter Enteral Nutr. 2016;40:159–211. 26773077 10.1177/0148607115621863

[32] Guan YC Tang YY Ma X. Advances in the study of glucocorticoid-induced hyperglycemia and diabetes mellitus. J Dalian Med Univ. 2022;44:157–62.

[33] Mo YZ Zhao F. Guidelines for perioperative glucose care in hyperglycemic patients. Chin J Nurs. 2017;52:794–8.

[34] Grau Carmona T López Martínez J Vila García B. Guidelines for specialized nutritional and metabolic support in the critically-ill patient: update. Consensus SEMICYUC-SENPE: respiratory failure. Nutr Hosp. 2011;26(Suppl 2):37–40. 10.1590/S0212-16112011000800008 22411517

